# The role of muscle forces on rotational and cranio‐caudal stability in the intact and CCL‐deficient stifle: An ex vivo biomechanical study

**DOI:** 10.1111/vsu.70122

**Published:** 2026-06-18

**Authors:** Pavlos Natsios, Rahel Capaul, Antonio Pozzi, Brian Park

**Affiliations:** ^1^ Clinic for Small Animal Surgery, Vetsuisse Faculty University of Zurich Zurich Switzerland; ^2^ Graduate School for Cellular and Biomedical Sciences University of Bern Bern Switzerland

## Abstract

**Objective:**

To investigate the effects of muscle activation on internal tibial rotation and cranio‐caudal translation (CCT) in intact and cranial cruciate ligament (CCL) deficient stifles.

**Study design:**

Ex vivo biomechanical study.

**Animals:**

Eight cadaveric, nonpaired canine stifles.

**Methods:**

Stifles were tested intact and after arthroscopic CCL transection. Quadriceps, biceps femoris, and gastrocnemius forces were simulated with pneumatic actuators in single or cocontraction muscle activation (0%–100% bodyweight [BW]). The tibia was mounted to a linear–torsional tester; the femur to a six‐degrees‐of freedom fixture. Internal tibial rotation (5 Nm torque) and CCT (30% BW cranial and caudal translation) were recorded via motion tracking system.

**Results:**

Without muscle activation, CCL transection increased internal tibial rotation (34.8 ± 11.8° CCL‐deficient vs. 27.9 ± 10.8° intact; *p* = .041). Across 0%–100% BW activation, internal rotation decreased in both conditions, to 4.3 ± 2.3° (intact; *p* < .0001 vs. 0% BW) and 2.8 ± 1.3° (CCL‐deficient; *p* = .001 vs. 0% BW) at 100% BW. At 100% BW, biceps femoris reduced internal rotation more than quadriceps and gastrocnemius in both CCL conditions. Muscle activation reduced CCT in CCL‐deficient stifles; however, at 100% BW, CCT was 19.1 mm in CCL‐deficient versus 2.8 mm in intact (+582%; *p* < .0001).

**Conclusion:**

Periarticular muscle activation mitigates axial plane rotational laxity but does not prevent CCT.

**Clinical significance:**

Targeted muscle strengthening may help manage rotational laxity; however, surgical stabilization remains necessary to address CCT after CCL rupture. Internal tibial rotation may require additional surgical stabilization in selected cases.

## 1 INTRODUCTION

The stifle joint is a complex synovial joint comprising the femorotibial and patellofemoral articulations.^1^, ^2^ Stifle motion occurs across three planes, reflecting the intricate anatomical relationships between static and dynamic stabilizers.^1^, ^2^ Static stabilizers include the ligaments, joint capsule, and menisci, while the geometry of the articular surfaces also contributes to joint congruency and passive stability.^1^, ^2^ Additionally, muscle forces and joint compression provide dynamic stability.^3^, ^4^ In a healthy stifle, these components function synergistically to maintain joint stability during movement.^5^ The cranial cruciate ligament (CCL) provides the primary passive restraint to internal tibial rotation and cranio‐caudal translation (CCT), whereas periarticular muscles—quadriceps, biceps femoris, and gastrocnemius—supply dynamic stability through compressive forces and reflex‐mediated activation.^3^, ^6^, ^7^

The role of muscle activation in joint loading and stability is critical in both dogs and humans.^6^, ^8^, ^9^, ^10^, ^11^, ^12^ In dogs, the hamstring muscles (biceps femoris, semitendinosus, and semimembranosus) have been reported to act as dynamic stabilizers: loading the semitendinosus in CCL‐deficient stifles reduces CCT, whereas quadriceps and gastrocnemius loading has been hypothesized to contribute to cranial tibial thrust in CCL‐deficient stifles under tibial compression/weightbearing configurations.^4^, ^13^ Dogs with spontaneous CCL rupture exhibit delayed hamstring reflexes, and brief quadriceps preactivation just before paw strike can partially curb cranial translation, underscoring the importance of precise muscle activation for stifle stability.^3^, ^14^ In humans, hamstring cocontraction reduces anterior tibial translation and anterior cruciate ligament (ACL) loading during weightbearing flexion, whereas quadriceps and gastrocnemius loading can increase anterior tibial shear/ACL load in an angle‐ and task‐dependent manner; antagonists’ cocontraction may also increase joint compression, which can enhance functional stability.^9^, ^10^, ^15^ Anatomically, the biceps femoris has prominent distal attachments on the lateral aspect of the proximal tibia, including a strong tendinous attachment to the lateral tubercle and a broad aponeurotic sheet extending distally over the tibia.^16^ Because these distal attachments are lateral, the biceps femoris line of action is expected to apply a caudolateral pull on the proximal tibia that can oppose excessive internal tibial rotation. This rationale is consistent with experimental cadaveric knee work showing that hamstrings cocontraction significantly reduces internal tibial rotation during weightbearing flexion,^9^ and that lateral hamstring loading can be more influential on tibiofemoral kinematics than medial hamstring loading.^17^

Internal‐external rotation kinematics in both intact and CCL‐deficient stifles depend on flexion angle and passive and active restraints. In the intact stifle, the tibia rotates internally approximately 20° relative to the femur during stifle flexion and returns to its initial position during extension.^18^, ^19^, ^20^ During normal stifle motion, the CCL primarily restrains internal tibial rotation near extension, whereas the cranio‐lateral joint capsule and the lateral collateral ligament provide the dominant restraint at greater flexion.^2^, ^18^, ^21^, ^22^ In the CCL‐deficient stifle, loss of the CCL as primary restraint leads to increased internal tibial rotation and greater reliance on secondary restraints, which are insufficient to prevent pathologic rotary laxity.^2^, ^18^, ^21^, ^22^ Excessive internal tibial rotation during stifle flexion contributes to pivot shift and rotational instability in both humans and canine patients.^23^, ^24^, ^25^ The role of muscle forces in controlling rotational instability in the CCL‐deficient stifle in dogs is poorly understood.

Our objective was to analyze the contributions of the quadriceps, biceps femoris, and gastrocnemius muscles to rotational and cranio‐caudal kinematics in the intact and CCL‐deficient stifle under weightbearing forces. We hypothesized that the biceps femoris would most effectively restrict internal tibial rotation, while the quadriceps femoris would have the greatest effect in limiting CCT in the CCL‐deficient stifle.

## 2 MATERIALS AND METHODS

Eight stifle joints (*n* = 8) were harvested from eight skeletally mature dogs, weighing between 20 and 40 kg, euthanized for reasons unrelated to this study. All cadaveric specimens were donated for research purposes, with written consent obtained prior to their use. Prior to testing, all specimens underwent computed tomography (CT) and magnetic resonance imaging (MRI) scanning to exclude any underlying stifle pathology that could influence the results. Stifles with concurrent pathologies or from chondrodystrophic breeds were excluded from the study. Immediately after euthanasia, the stifles were harvested, and specimens were wrapped in saline‐soaked towels and stored at −20°C until testing. The limb (either left vs. right) from each donor included in the study was selected using a random number generator to prevent systematic laterality bias.

### 2.1 Specimen preparation

**FIGURE 1:**
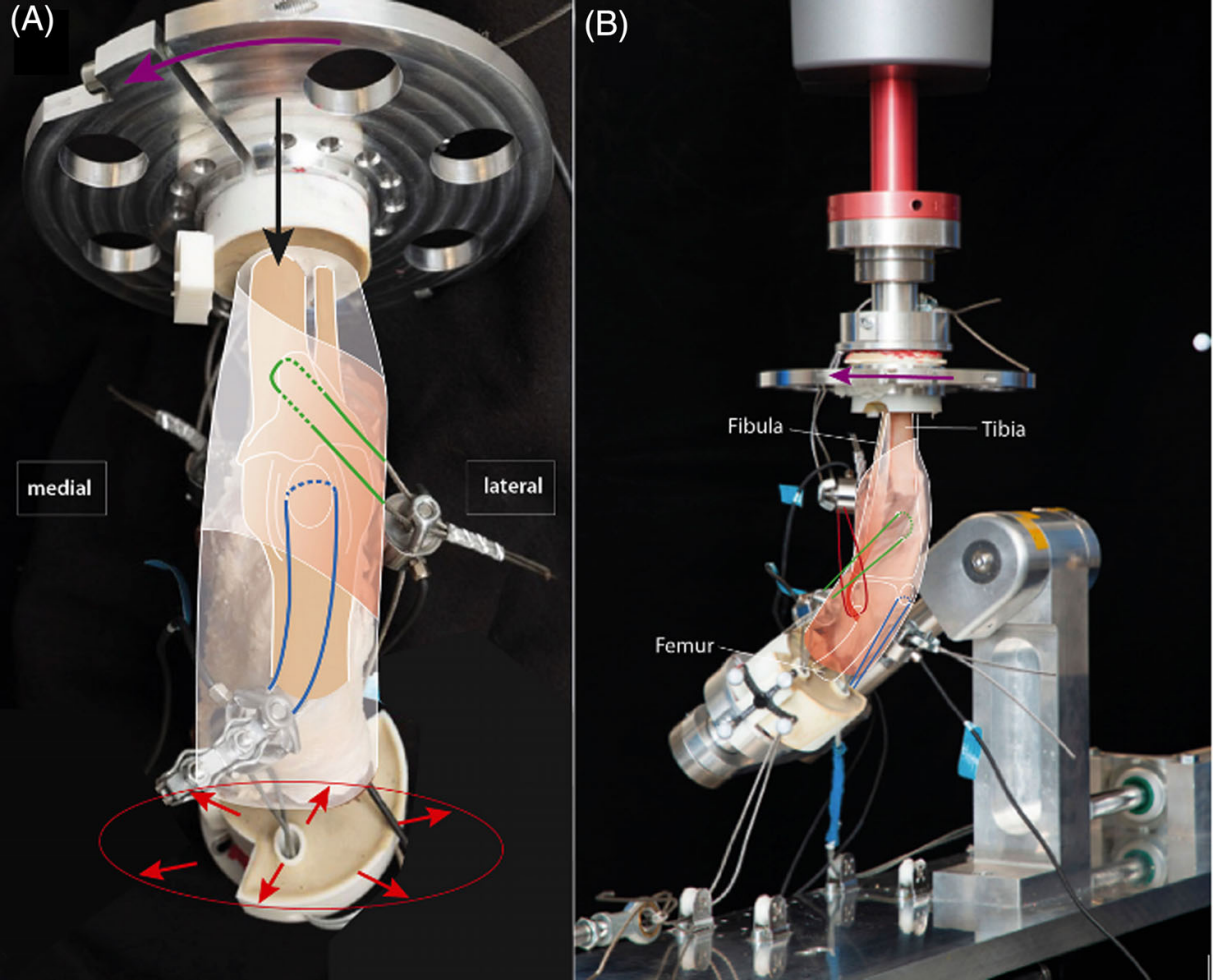
Femoral/tibial fixtures and cable routing used for muscle‐force simulation. (A) Close‐up view of the potted stifle with simplified overlays showing the cable paths used to simulate the quadriceps (blue) and biceps femoris (green). Medial is to the left and lateral is to the right. Loading/constraint directions are indicated in this panel: Axial compressive load (30% bodyweight; black arrow), imposed internal tibial rotation (purple arrow), and translational degrees of freedom permitted at the femoral base (red arrows). Dashed segments indicate portions of the cable path that are not visible in this view (e.g., passing deep/within the construct). (B) Overview of the stifle mounted in the linear–torsional testing system showing the femoral and tibial fixtures and the color‐coded cable routing used to simulate the quadriceps (blue), biceps femoris (green), and gastrocnemius (red). The direction of imposed internal tibial rotation is indicated (purple arrow). Muscle forces were applied using in‐line actuators and measured with in‐line load cells.

Prior to testing, the specimens were thawed at room temperature for 24 h and prepared for mechanical testing. Soft tissues at the distal tibia and proximal femur were dissected to facilitate fixation in the testing machine, while the periarticular soft tissues within 5 cm proximal and distal to the stifle were preserved. The femur of each specimen was potted in a custom‐made, three‐dimensional (3D)‐printed plastic mold using polymethylmethacrylate (PMMA). The femoral mold included ducts to allow passage of wires simulating the pull of the biceps femoris and quadriceps muscles, replicating physiologic lines of action. The distal tibia was potted in an acrylic cylinder (60 mm diameter, 50 mm length) using PMMA. A custom‐made disk was mounted on the potted cylinder to rigidly attach a pneumatic actuator used for muscle force application (Figure 1).

### 2.2 Muscle force simulation

The muscle forces of the quadriceps, biceps femoris, and gastrocnemius muscles were simulated using pneumatic actuators (Festo AG, Zurich, Switzerland) linked to 2 mm metal wire cords fixed at their anatomical attachment sites via bone tunnels. For the quadriceps, a 2.5 mm drill bit was used to create a mediolateral hole through the patella, perpendicular to its long axis, to transmit force along the patellar ligament. The biceps femoris cable was secured through two mediolateral bone tunnels (2.5 mm diameter) in the tibial tuberosity at the level of the patellar ligament insertion, with a 10–15° proximal angulation on the lateral side to reproduce its physiologic line of action. The gastrocnemius was simulated by routing a cable caudal to the femoral condyles around the fabelae, reproducing its native course across the stifle (Figure 1). In‐line load cells with a digital display were attached between the bones and the pneumatic actuators to monitor and adjust muscle forces in real time.

### 2.3 Biomechanical testing

**FIGURE 2:**
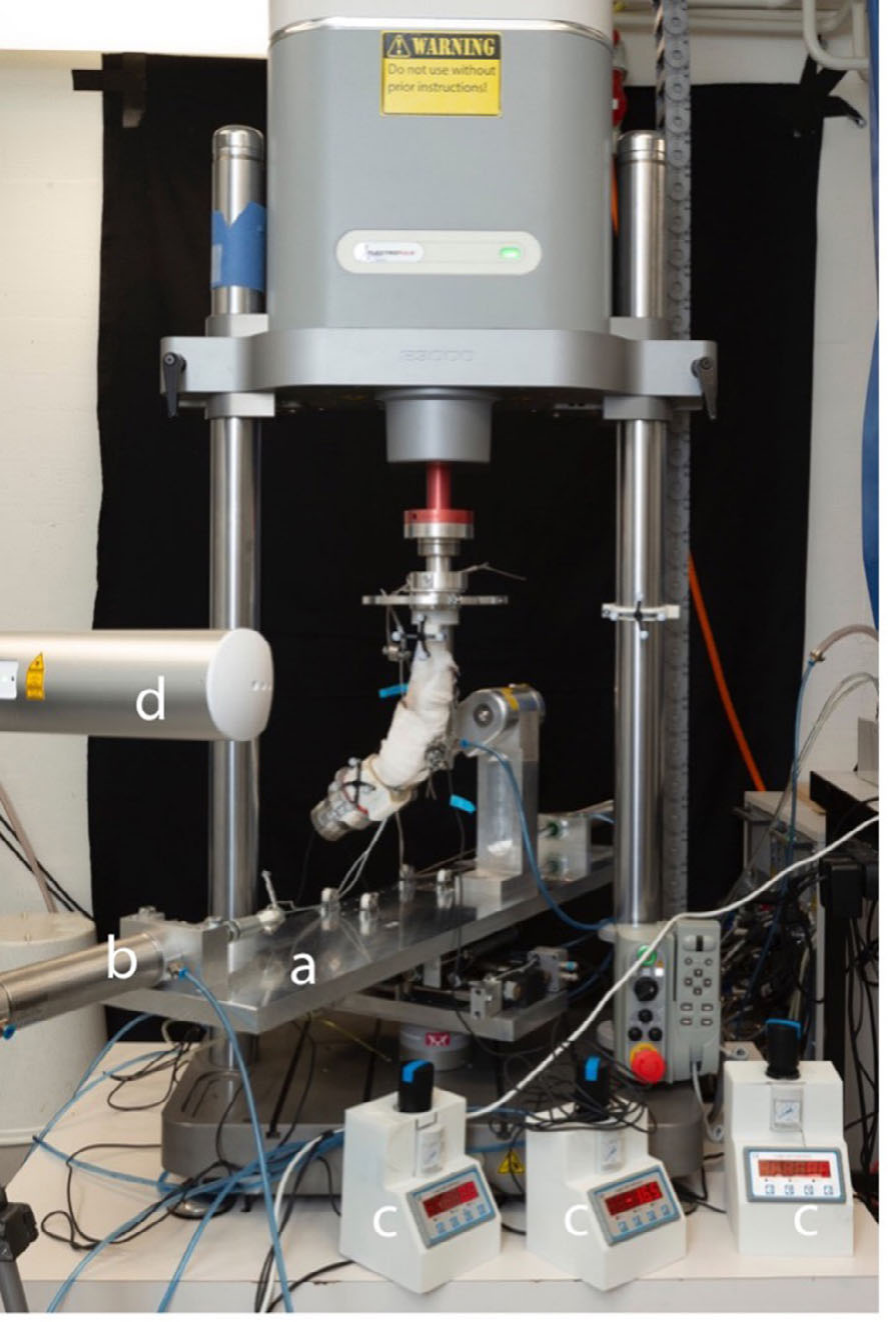
Experimental setup. The distal tibia is potted in an acrylic cylinder and rigidly coupled to the rotary/linear actuator of a linear–torsional electromechanical testing system (Instron E3000, Norwood, Massachusetts). (A) The proximal femur is secured in a custom six‐degrees‐of‐freedom fixture mounted to the base, permitting self‐alignment while minimizing constraint artifacts. (B) Muscle loads are generated by pneumatic actuators which are (C) controlled by digital pressure regulators and transmitted via stainless steel cables routed to reproduce the physiologic lines of action of the quadriceps, gastrocnemius, and biceps femoris; cable forces are monitored with in‐line load cells. For the 0% activation condition, all cables are set slack (0 N). A base‐mounted pneumatic actuator applies cranio‐caudal shear for cranio‐caudal translation (CCT) testing, and the test machine applies axial compression (30% bodyweight). (D) An optical motioncapture system (FusionTrack 500, Atracsys SA, Puidoux, Switzerland; 100 Hz) tracks reflective marker clusters on the femur and tibia to compute internal tibial rotation (°) and cranio‐caudal translation (CCT, mm).

**FIGURE 3:**
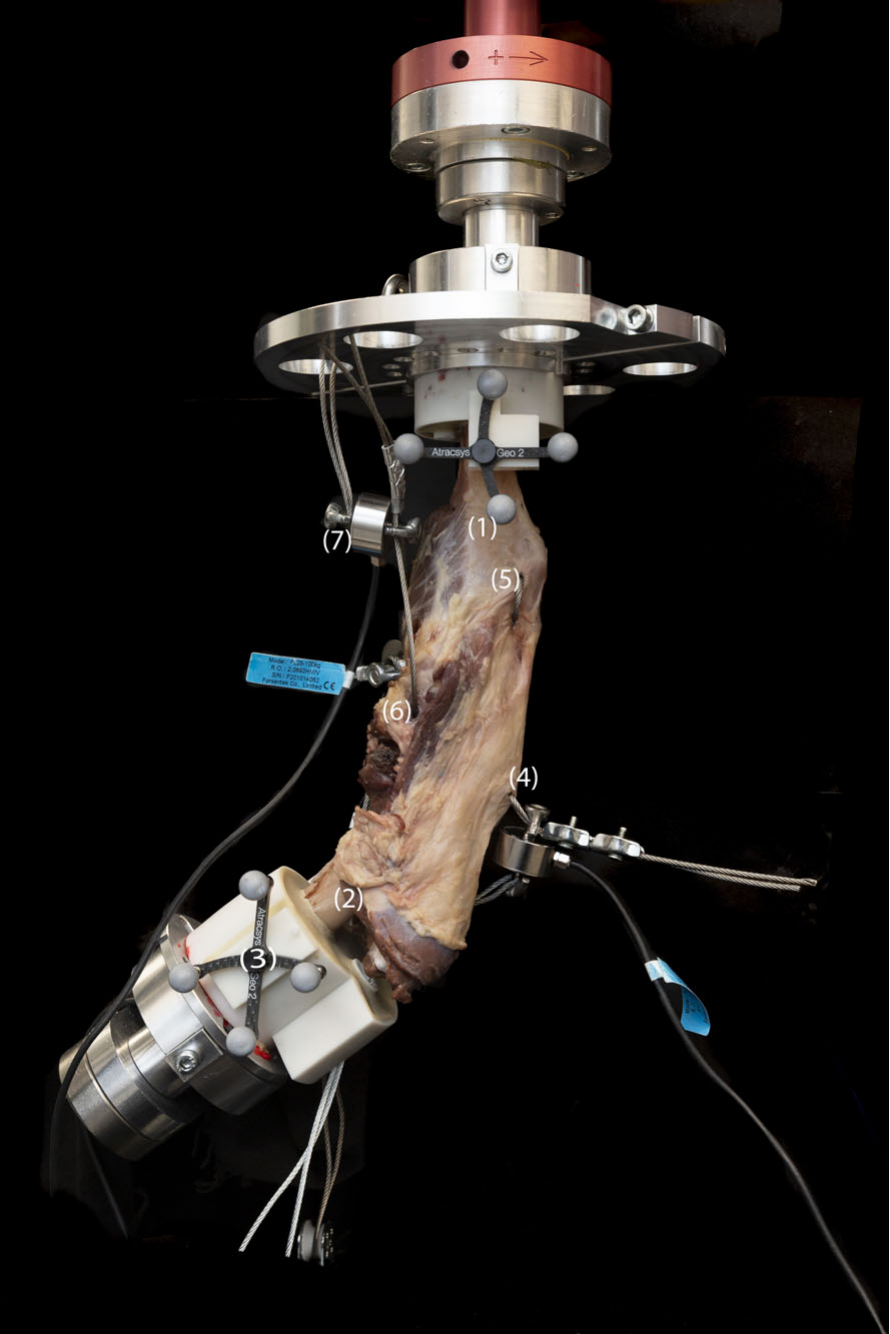
Experimental setup (right stifle; lateral view). The tibia is potted and aligned with the rotary axis of a linear–torsional testing system (Instron E3000) (**1**); the femur is potted and secured in a custom six‐degree‐of‐freedom fixture (**2**). Reflective marker clusters on the femur and tibia (**3**) enable optical tracking (FusionTrack 500, 100 Hz) of internal tibial rotation (°) and cranio‐caudal translation (CCT, mm). Muscle loads are applied by pneumatic actuators (off frame) and transmitted via stainless‐steel cables routed along physiologic lines of action: Quadriceps via a mediolateral patellar tunnel (**4**), biceps femoris anchored at the tibial tuberosity with lateral 10–15° proximal angulation (**5**), and gastrocnemius routed caudal to the femoral condyles around the fabellae (**6**); inline load cells (**7**) monitor cable forces. The machine applies axial compression (30% bodyweight); a base‐mounted shear actuator (not visible) applies cranio‐caudal loading for CCT tests.

For the mechanical testing, the tibia was fixed to the actuator of a linear‐torsion all‐electric mechanical testing machine (Instron E3000, Norwood) to apply controlled compression and rotational forces. Each tibia was aligned to the rotational center of the machine to ensure that forces and moments were applied along the long axis. The femur was secured to a custom‐made fixture that allowed 6° of freedom (Figures 1 and 2). A pneumatic actuator was attached to the base tray to apply cranio‐caudal loads, specifically for testing CCT. Each stifle was positioned at a 135° flexion angle, and reflective motion‐tracking markers were placed on the distal tibia and proximal femur. CCT data were collected using a motion tracking system (Fusion Track 500, Atracsys LLC, Puidoux, Switzerland) (Figure 3). The custom‐made 6‐degrees‐of‐freedom system, combining the Instron E3000 testing machine, load cells, and pneumatic actuator, has been validated for spinal biomechanical testing and adapted to the stifle.^26^, ^27^ The intact stifles were tested first and served as the control for comparison with the CCL‐deficient measurements.

During testing, muscle‐specific loads were applied to simulate single muscle contraction or cocontraction combinations of the quadriceps, gastrocnemius, and biceps femoris. Measurements were taken under increasing loads from 0%–100% of bodyweight (BW) at 0%, 20%, 30%, 40%, 60%, 80%, and 100% of BW for both internal rotation and CCT in intact and CCL‐deficient stifles (Table 1). The wires had no tension during the testing with no muscle activation. The experimental setup was designed to ensure that all cables were pulled in a physiologic, muscle‐specific line of action, with guide channels incorporated into the potting fixtures to ensure correct alignment for the quadriceps and biceps femoris (Figure 1). Subsequently, after the muscle force activation, all stifles were subjected to an axial force (10 N/s) equivalent to 30% of the specimen's BW to simulate a midstance weightbearing condition.^28^ To test internal rotation, the tibia was rotated under displacement control at a speed of 10°/s until a torque load of 5 Nm was achieved. The maximum internal rotation angle was recorded at 100 Hz.^29^ For CCT testing, CCT was simulated under load control by applying a force equivalent to 30% of the specimen's BW in the cranial and caudal directions. The test was repeated three times, and the motion capture system was used to track CCT with a sampling frequency of 100 Hz. The maximum cranial displacement was extracted for analysis.

**TABLE 1:** Testing matrix applied to each specimen. All stifles were tested at 135° flexion under 30% BW axial compression.

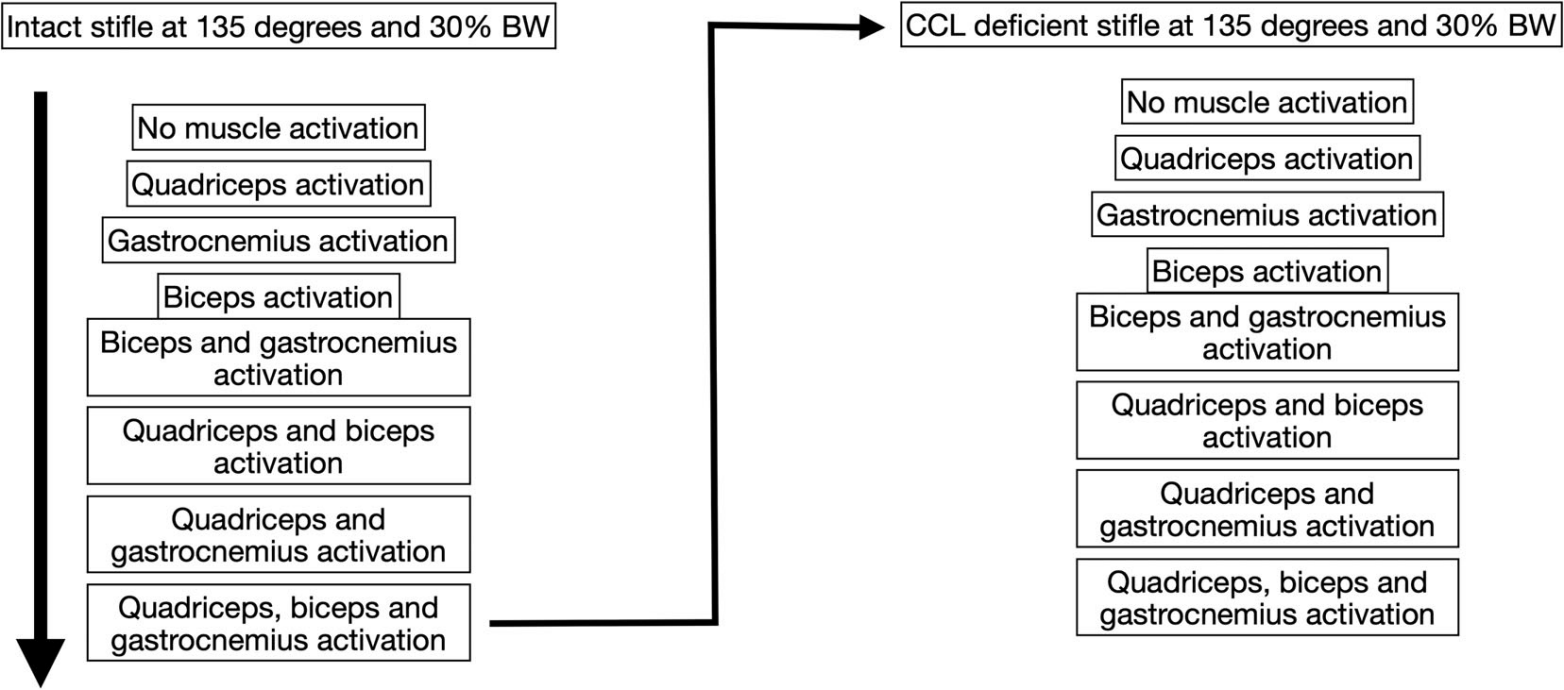

*Note*: For each stifle, the matrix comprised: Baseline (no muscle activation); single muscle conditions (quadriceps, biceps femoris, gastrocnemius); two muscle cocontractions (quadriceps + biceps femoris; quadriceps + gastrocnemius; biceps femoris + gastrocnemius); and three muscle cocontraction (quadriceps + biceps femoris + gastrocnemius). Each condition was tested at seven activation levels: 0%, 20%, 30%, 40%, 60%, 80%, and 100% BW. The entire matrix was completed in the intact stifle and then repeated after arthroscopic CCL transection.

Abbreviations: BW, bodyweight; CCL, cranial cruciate ligament.

All measurements of the intact stifle were used as control data. Then, the CCL was arthroscopically transected through a single medial parapatellar port, ensuring no other passive stabilizing structures were compromised. The CCL transections were performed while the specimens were still mounted on the mechanical testing machine to eliminate any variances that might have resulted from the dismounting and remounting process. The same testing protocol was then repeated in the CCL‐deficient stifles (Table 1). All measurements were performed beginning from the specimen's neutral position, with no deviation.

### 2.4 Statistical analysis

A sample size of eight stifles was chosen, following a sample size consistent with values commonly reported in comparable ex vivo canine stifle studies.^28^, ^30^

For both rotation and translation data, means ± SD were calculated for all conditions. Statistical analyses were performed using SPSS (IBM Corp., Armonk, New York). Rotation and translation data were evaluated separately. To assess the overall effects of muscle condition (quadriceps, gastrocnemius, biceps femoris, and cocontraction), activation level (20%–100% BW), and stifle condition (intact vs. CCLR), a three‐way repeated‐measures ANOVA was conducted. Prior to analysis, rotation data were square‐root transformed and translation data were reciprocal square‐root transformed to reduce skewness and heteroscedasticity, improving the distribution of residuals. No spurious or influential observations were detected following transformation (all standardized residuals < ±3). Normality of residuals was assessed both statistically (Shapiro–Wilk test) and visually (Q–Q plot). Despite minor deviations detected by the Shapiro–Wilk test (*p* < .05), Q‐Q plots demonstrated that residuals were approximately normal after transformation. Levene's test and spread versus level plot confirmed homogeneity of variance between groups, and Mauchly's test of sphericity was performed for within‐subject factors; when violated, Greenhouse–Geisser corrections were applied. No outliers were identified after transformation, and Bonferroni adjustments were applied.

To evaluate the effect of increasing muscle activation relative to the 0% (passive condition) and to identify potential plateaus in response, additional pairwise within‐subject comparisons were performed between the 0% and each load activation level (20%, 30%, 40%, 60%, 80%, and 100% BW). Because the 0% condition was not included in the repeated‐measures ANOVA, these comparisons were conducted separately using paired‐samples *t*‐tests. Analyses were performed independently for each muscle group and knee condition (intact and CCL‐deficient) to determine the activation threshold at which joint rotation differed significantly from the passive state. Bonferroni adjustments were applied for multiple pairwise comparisons. A *p*‐value <.05 was considered statistically significant for all analyses.

## 3 RESULTS

The specimens were from skeletally mature dogs, with a mean weight of 28.4 kg (range: 25.0–38.5 kg). Eight non‐paired stifles were included (*n* = 8), comprising five right hindlimbs and three left hindlimbs. The specimen population consisted of mixed non‐chondrodystrophic breeds with no stifle pathology. The cohort comprised Labrador Retrievers (*n* = 3), a Standard Poodle (*n* = 1), a German Shepherd (*n* = 1), mixed‐breed dogs (*n* = 2), and a Weimaraner (*n* = 1).

### 3.1 Internal tibial rotation

**FIGURE 4:**
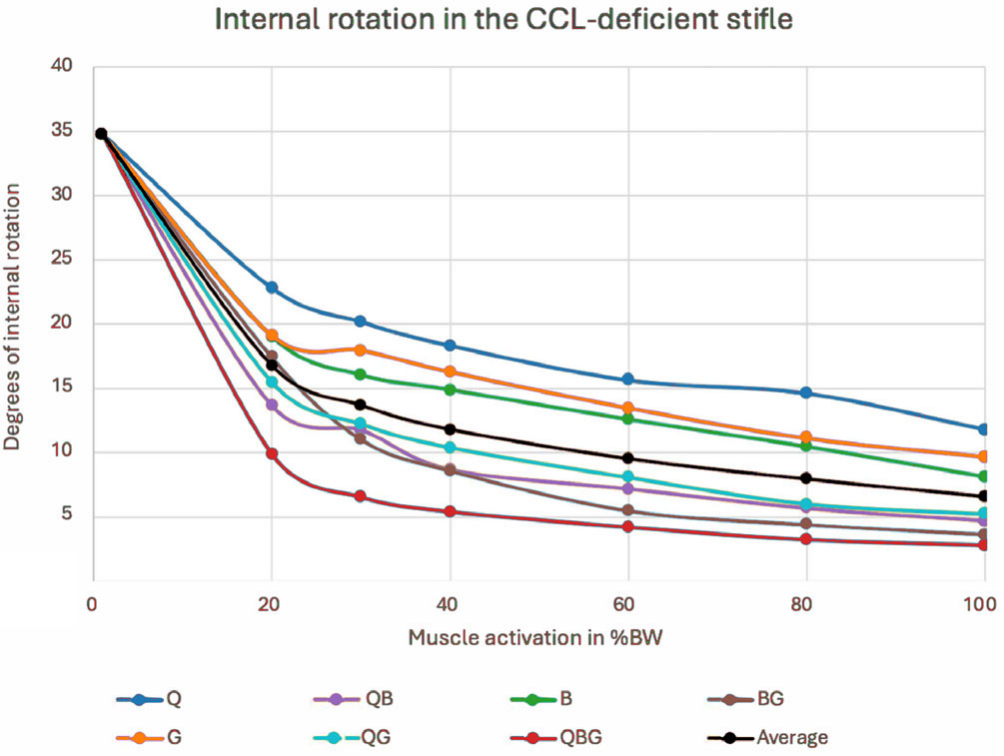
Internal tibial rotation with muscle activation in cranial cruciate ligament (CCL)‐deficient stifles (*n* = 8). Mean internal tibial rotation (°) across activation levels 0%–100% bodyweight (BW) for single‐muscle conditions—quadriceps (Q), biceps femoris (B), gastrocnemius (G)—and cocontractions—quadriceps + biceps femoris (QB), quadriceps + gastrocnemius (QG), biceps femoris + gastrocnemius (BG), and three‐muscle (QBG). Line colors correspond to the in‐figure legend.

The mean internal rotation of the intact stifle without muscle activation was 27.9° ± 10.8° (transformed value: 5.24° ± 0.68°). In the CCL‐deficient stifle, internal rotation increased to 34.8° ± 11.8° (transformed value: 5.8° ± 0.65°; *p* = .041). In the intact stifle, the mean internal tibial rotation decreased from 27.9° ± 10.8° to 4.3° ± 2.3° with 100% BW muscle activation (transformed value: 5.24° ± 0.68° to 2.01° ± 0.5°; *p* < .0001). A similar trend was observed in the CCL‐deficient stifle, where internal rotation decreased from 34.8° ± 11.8° to 2.8° ± 1.3° (transformed value: 5.8° ± 0.65° to 1.65° ± 0.34°; *p* = .001) (Figure 4). Across increasing muscle activation levels, internal rotation decreased significantly in both intact and CCL‐deficient stifles (Table 2). At 100% BW under three muscle cocontraction, internal tibial rotation did not differ significantly between intact and CCL‐deficient stifles (4.3 ± 2.3° vs. 2.8 ± 1.3°, *p* > .05).

**TABLE 2:** Percent change in the capacity to limit internal rotation in intact versus CCL deficient stifles (CCLD).

Muscle activation (%BW)	Q		B		G		QBG	
	Intact	CCLD	Intact	CCLD	Intact	CCLD	Intact	CCLD
20	12.4%	31.1%*	14.4%	45.0%*	13.2%	44.4%*	55.3%	68.4%
30	20.8%	39.0%*	20.5%	54.4%*	22.2%	48.0%*	66.3%	79.7%
40	30.3%	44.6%	32.6%	57.9%*	29.6%	52.9%*	70.8%	83.2%
60	36.3%	52.6%	44.1%	64.4%	38.8%	60.9%*	78.7%	87.0%
80	42.8%	55.9%*	51.9%	70.4%	47.9%	67.9%*	80.2%	89.8%*
100	46.7%	64.0%*	65.5%	76.9%	53.3%	72.0%*	84.6%	91.3%*

*Note*: Values are expressed relative to each stifle's noactivation baseline (0% BW load) at muscle loads of 20%–100% BW. Columns report singlemuscle activations—quadriceps (Q), biceps femoris (B), gastrocnemius (G)—and the threemuscle cocontraction (QBG). Positive values indicate a greater reduction in internal rotation compared with baseline (i.e., greater stabilizing effect).

Abbreviations: B, biceps femoris; BW, bodyweight; CCL, cranial cruciate ligament; CCLD, CCL deficient; G, gastrocnemius; Q, quadriceps; QBG, quadriceps–biceps femoris–gastrocnemius cocontraction.

* Significantly different from the corresponding intact value at the same load (*p* < .05).

**FIGURE 5:**
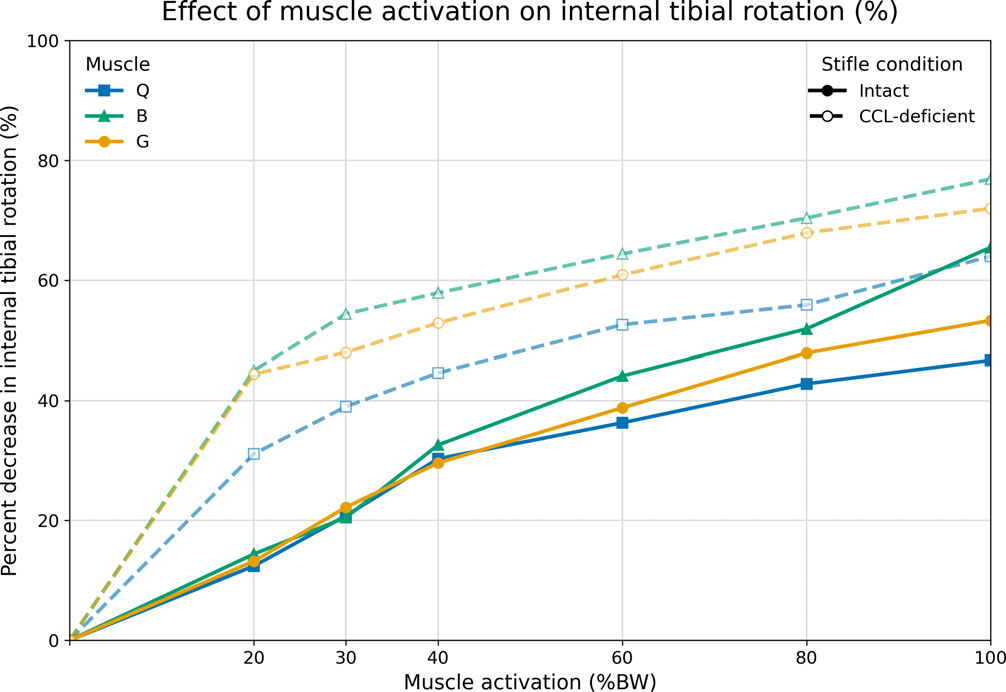
Percentage decrease in internal tibial rotation (IR) with single muscle activation in intact and cranial cruciate ligament (CCL)‐deficient stifles (*n* = 8). Curves show the mean percentage decrease relative to the within‐condition baseline (0% activation of all muscles), computed as [(IR₀ − IRload)/IR₀] × 100. Data are shown for quadriceps (Q), biceps femoris (B), gastrocnemius (G) at activation levels 0%, 20%, 30%, 40%, 60%, 80%, 100% bodyweight (BW); colors/markers correspond to the in‐figure legend.

At 100% BW in the intact stifle, isolated biceps femoris activation resulted in significantly less internal rotation (9.9° ± 6.6°; transformed value: 3.03° ± 0.95°) compared with isolated quadriceps (*p* = .016) and isolated gastrocnemius (*p* = .007). However, between 20% and 80% BW activation, there was no significant difference in internal rotation between the single muscle conditions (Figure 5). In the CCL‐deficient stifle, at 100% BW activation, the biceps femoris resulted in 8.1° ± 4.1° (transformed value: 2.81° ± 0.88°) of internal rotation, with no significant difference from the gastrocnemius. However, a significant difference between the biceps femoris and quadriceps emerged at ≥80% BW (80%: *p* = .009; 100%: *p* = .038).

### 3.2 Cranio‐caudal translation (CCT)

**FIGURE 6:**
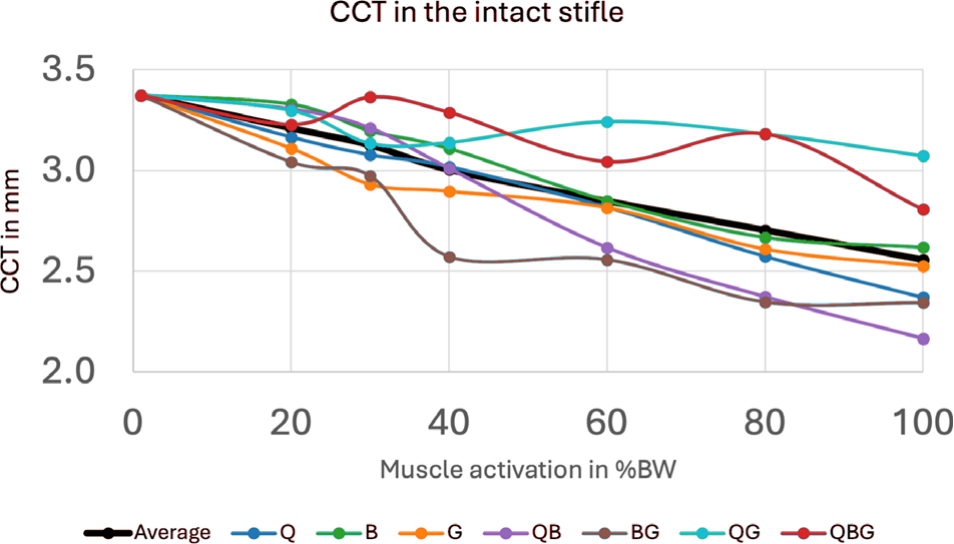
Cranio‐caudal translation (CCT) in the intact stifle across muscle activation levels (*n* = 8). Curves show mean CCT (mm) at 0%, 20%, 40%, 60%, 80%, and 100% bodyweight (BW) for isolated muscles—quadriceps (Q), biceps femoris (B), gastrocnemius (G)—and cocontractions (QB, QG, BG, QBG). The “average” trace denotes the mean of all muscle conditions at each load.

**FIGURE 7:**
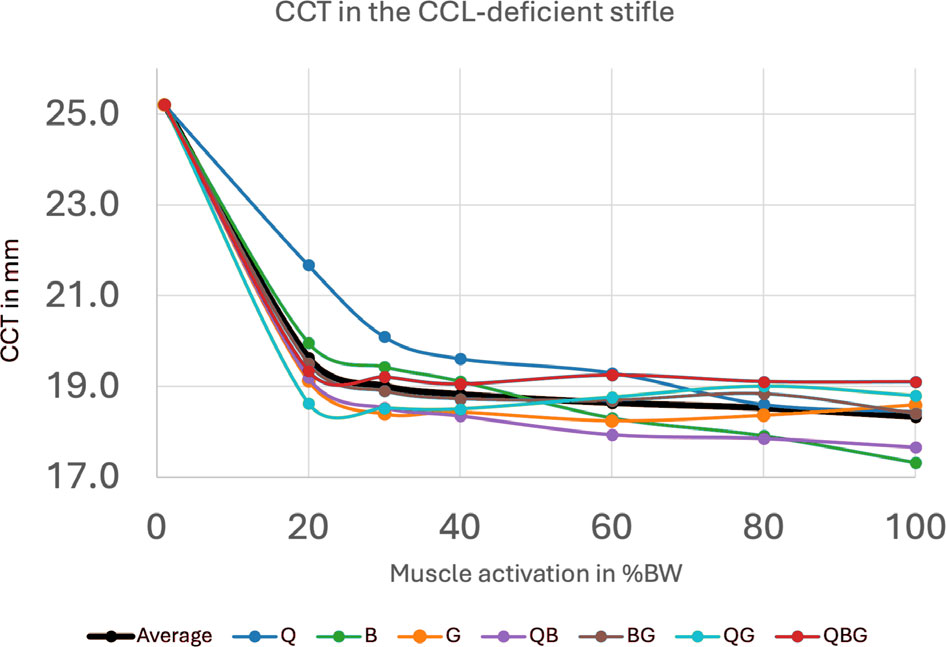
Cranio‐caudal translation (CCT) in cranial cruciate ligament (CCL)‐deficient stifles across muscleactivation levels (*n* = 8). Curves show mean CCT (mm) for isolated quadriceps (Q), biceps femoris (B), gastrocnemius (G) and cocontractions (QB, QG, BG, QBG) at 0%, 20%, 40%, 60%, 80%, 100% bodyweight (BW); the “average” trace is the mean across all muscle conditions at each load.

In intact stifles, the reduction in CCT from 0% to 100% BW under three muscle cocontraction was modest and not statistically significant (3.4 ± 0.5 mm vs. 2.8 ± 0.5 mm; transformed value: 0.55 ± 0.05 mm vs. 0.60 ± 0.05 mm, *p* > .05). In CCL‐deficient stifles, CCT decreased from 25.2 ± 2.4 mm at 0% to 19.1 ± 1.2 mm at 100% BW (transformed value: 0.20 ± 0.01 mm to 0.23 ± 0.01 mm, *p* < .0001) (Figures 6 and 7). At 100% BW activation, the CCL‐deficient stifle (mean ± SD, 19.1 ± 1.2 mm, transformed value: 0.23 ± 0.01 mm) exhibited approximately 6.8‐fold higher CCT than the intact stifle (mean ± SD, 2.8 mm ± 0.5 mm, transformed value: 0.60 ± 0.05 mm) (*p* < .0001).

At 100% BW in the CCL‐deficient stifle, CCT was 17.3 ± 1.7 mm for the biceps femoris (transformed value: 0.24 ± 0.01 mm), with no significant differences between muscles (biceps vs. quadriceps, *p* = .39; biceps vs. gastrocnemius, *p* = .42). A significant reduction in CCT relative to 0% activation was observed for all singlemuscle conditions, from 20% BW for the gastrocnemius and biceps femoris and from 40% BW for the quadriceps; however, the absolute magnitudes of CCT remained large and far above intact values, and meaningful normalization of sagittal plane stability was not achieved.

Complete numerical results for all activation levels and muscle conditions are provided in Table S1 (internal rotation) and Table S2 (cranio‐caudal translation)‐Supporting Information.

## 4 DISCUSSION

In this ex vivo stifle model with simulated muscle and weightbearing forces, the CCL‐deficient stifle exhibited increased internal rotation and CCT compared to intact limbs in the absence of muscle activation. In the axial plane, simulated muscle activation markedly reduced internal tibial rotation in the CCL‐deficient stifle; at 100% BW, three‐muscle cocontraction, internal rotation was not significantly different from that of the intact. In contrast, muscle activation failed to restore cranio‐caudal alignment. In the sagittal plane, three muscle cocontraction reduced cranial tibial translation after CCL transection, but translation remained ~6.8‐fold higher than intact at 100% BW, indicating partial mitigation rather than restoration of stability. Although the biceps femoris contributed notably to controlling internal tibial rotation, it was ineffective in significantly reducing CCT. Our results confirm that muscle forces are insufficient to neutralize femorotibial subluxation, without the contribution of a surgical stabilization.

Unlike earlier passive cadaver studies, which either examined isolated muscle contributions^13^ or relied on theoretical simulations of periarticular muscle forces,^31^ our testing protocol simultaneously activated the quadriceps, gastrocnemius, and biceps femoris under a midstance, weightbearing condition with graded cocontraction. Prior ex vivo models using fixed cables or turnbuckles to simulate muscles reported cranial tibial translations of about 10–20 mm after CCL transection.^28^, ^32^ The translations observed in our model fell within this range, indicating that substantial instability persists in the CCL‐deficient stifle despite active muscle engagement. Unlike the models with fixed‐tension, our testing set up allows to modulate muscle tension and simulate various protocols, offering a more accurate understanding of how active muscular forces influence stifle stability.

In the sagittal plane, muscle loading could not fully neutralize CCT in CCL‐deficient stifles.^22^ Even at 100% BW muscle activation, CCT remained about 582% higher than in intact stifles, indicating that muscle force alone cannot compensate for CCL loss. Our results align with ex vivo studies that suggested a modest effect of quadriceps tension on reducing tibial translation,^13^, ^14^ without normalizing stifle stability. Indeed, the limited effectiveness of muscle forces to fully restrain CCT confirms that the CCL is the essential primary restraint in the sagittal plane.^13^, ^14^, ^22^, ^33^ Mechanistically, biceps femoris best limited internal tibial rotation because its lateral distal tibial attachments generate an external‐rotation moment, whereas quadriceps and gastrocnemius act mainly in the sagittal plane and primarily increase tibiofemoral compression rather than providing a directed caudal shear to counter cranial thrust; thus, muscle loading may modestly reduce drawer displacement but cannot replace the ligament. By contrast, intact stifles displayed only minimal CCT under the same load (~3 mm), reflecting the CCL's primary role in resisting cranial thrust.^21^

Isolated gastrocnemius activation reduced cranio‐caudal translation in our load‐controlled drawer test. This finding should be interpreted in the context of a non‐weight bearing test mode and not conflated with gastrocnemius‐associated cranial tibial thrust described under tibial compression/weight‐bearing conditions. Gastrocnemius tension can contribute to cranial tibial thrust in CCL‐deficient stifles under certain limb configurations, which depend on distal limb/tarsal mechanics and joint position.^13^, ^34^, ^35^ However, cocontraction of the quadriceps and gastrocnemius muscles acts as a knee stabilizing mechanism in people during walking.^34^ Accordingly, the observed reduction in CCT should not be interpreted as evidence that gastrocnemius reduces cranial tibial thrust in vivo. Importantly, the effect was modest and non‐normalizing, with CCL‐deficient translation remaining substantially greater than intact values even at the highest activation levels.

This study demonstrates that simulated muscle activation provides only partial stabilization of the CCL‐deficient stifle giving insights on surgical and conservative treatment of CCL disease. Based on these results, targeted muscle strengthening and neuromuscular training should be recommended in combination with surgical stabilization. Inadequate timing or imbalance of muscular activation may increase the risk of secondary joint damage and contribute to accelerated osteoarthritis.^35^, ^36^ Because early postoperative muscle activation is minimal, due to chronic CCL disease and to the surgical procedure itself, surgical stabilization remains necessary to address cranial tibial thrust, and adjunct approaches aimed at improving axial‐plane stability should be considered in selected cases, while rehabilitation progressively restores dynamic stability.

The results of the rotational range of motion are consistent with findings from in vivo studies.^18^, ^22^ Intact cadaveric stifles exhibited slightly greater internal rotation than typically reported in vivo, likely reflecting the absence of active muscle tone/neuromuscular control and differences in periarticular soft tissue tension. CCL transection produced a further increase in internal rotation, mirroring in vivo kinematic studies of naturally occurring disease.^4^, ^22^, ^37^ The increased internal rotation observed in the current study aligns with the known role of the CCL in restraining excessive tibial rotation at mid‐stance flexion angles.^21^ These findings also dovetail with the stifle's screw home mechanism. In the intact stifle, graded activation of periarticular muscles likely augments the effect of articular geometry and ligament tension via joint compression and caudally directed moments,^9^ which helps explain the large, load‐dependent reductions in internal rotation we observed. By contrast, CCL transection disrupts this coupling, which cannot be fully normalized by muscle activation alone.

When translating our findings to clinical decision‐making, we should consider that alterations in neuromuscular patterns are still detectable at ~12 months after ACL reconstruction in humans.^38^ Neuromuscular activation deficits can remain substantial even >3 years post‐reconstruction, reinforcing the concept that the contribution of muscle activation to rotational stability may not be effective early after surgery.^39^ In this context, lateral extra‐articular augmentation combined with TPLO and ACL reconstruction in dogs and people, respectively, are a mean to temper residual rotational laxity while muscles are strengthened during postoperative rehabilitation. Data from studies in dogs and people show that adding anterolateral procedures—anterolateral ligament reconstruction or lateral extra‐articular stabilization—to ACL reconstruction and TPLO, improves the control of tibial internal rotation and the pivot shift under pivot‐type loading.^40^, ^41^, ^42^

This study has inherent limitations due to its ex vivo, cadaver‐based model. The model simulates mid‐stance loading but cannot reproduce the multidirectional, cyclic behavior of a living stifle. In addition, testing was performed at a single joint angle, which may limit extrapolation of these findings across the full range of stifle motion, where ligament restraint and muscle contributions to stability may vary. We evaluated only three muscles (quadriceps, biceps femoris, gastrocnemius); contributions from other muscles, native cocontraction patterns, and neuromuscular timing were not modeled. Notably, the semitendinosus muscle was not tested, which represents a limitation given its documented role in reducing cranial tibial translation in CCL‐deficient stifles.^13^ Because muscle forces were set before axial loading, small inconsistencies in force distribution may have occurred. Activation magnitudes were discretized (0%–100% BW in 20% steps) and cable‐driven surrogates only approximate in vivo forces; future work should incorporate feedback‐controlled actuation and EMG‐informed profiles to improve translational relevance. Furthermore, we did not include a TPLO‐stabilized condition (with or without lateral augmentation); therefore, we cannot directly assess how TPLO influences rotational laxity or cranio‐caudal stability under our specific testing conditions. Finally, because CCT and internal rotation were measured in separate tests, we did not quantify coupled translation during internal rotation (pivot‐type loading); therefore, the implications of periarticular muscle activation for pivot‐shift behavior in vivo remain to be clarified.

This study provides insights into the role of muscle activation on joint stability in the CCL‐deficient stifles. Among the individual muscles tested, the biceps femoris was the most effective at limiting internal tibial rotation, while the lowest internal rotation overall occurred with three‐muscle cocontraction. In contrast, none of the tested muscles, alone or in cocontraction, restored cranio‐caudal stability after CCL transection, and substantial CCT persisted even at maximal activation. These findings suggest that surgical stabilization is essential to effectively address CCT and support the use of targeted muscle strengthening, particularly of the biceps femoris, to manage rotational instability. The results presented here establish a foundation for future biomechanical studies investigating dynamic muscular contributions to canine stifle stability, and may help refine integrated surgical and rehabilitative strategies for clinical management of CCL‐deficient stifles.

## AUTHOR CONTRIBUTIONS

Natsios P, DVM, MSc., PhD, DECVS: Writing – review and editing, writing – original draft, visualization, validation, project administration, methodology, investigation, formal analysis, data curation and conceptualization. Capaul R, DVM: Writing – review and editing, writing – original draft, visualization, project administration, investigation, formal analysis, and data curation. Pozzi A, DVM, MS, DACVS (Small Animal) , DECVS, DACVSMR: Writing – review and editing, writing – original draft, visualization, validation, methodology, and conceptualization. Park B, PhD: Writing – review and editing, writing – original draft, visualization, validation, project administration, methodology, investigation, formal analysis, data curation, and conceptualization.

## FUNDING INFORMATION

The authors declare that no financial support was received for the research, authorship, and/or publication of this article.

## CONFLICT OF INTEREST STATEMENT

The authors declare that the research was conducted in the absence of any commercial or financial relationships that could be construed as a potential conflict of interest.

## Supporting information


**Table S1.** Tibial internal rotation in degrees for the intact and CCL‐deficient stifle (±SD) at different muscle groups and activation levels with increments of 20% BW.
**Table S2.** Mean CCT in the intact and CCL‐deficient stifle (±SD) at graded muscle activation increments of 20% BW.

## References

[1] Miller's Anatomy of the Dog. Elsevier Saunders; 2013.

[2] Vasseur PB , Arnoczky SP . Collateral ligaments of the canine stifle joint: anatomic and functional analysis. Am J Vet Res. 1981;42(7):1133‐1137. 7271030

[3] Hayes GM , Granger N , Langley‐Hobbs SJ , Jeffery ND . Abnormal reflex activation of hamstring muscles in dogs with cranial cruciate ligament rupture. Vet J. 2013;196(3):345‐350. doi:10.1016/j.tvjl.2012.10.028 23219226

[4] Korvick DL , Pijanowski GJ , Schaeffer DJ . Three‐dimensional kinematics of the intact and cranial cruciate ligament‐deficient stifle of dogs. J Biomech. 1994;27(1):77‐87. doi:10.1016/0021-9290(94)90034-5 8106538

[5] Nishizawa Y , Tashman S . In vivo biomechanics: laxity versus dynamic stability. In: Musahl V , Karlsson J , Kuroda R , Zaffagnini S , eds. Rotatory Knee Instability. Springer International Publishing; 2017:37‐48. doi:10.1007/978-3-319-32070-0_4

[6] Solomonow M , Baratta R , Zhou BH , et al. The synergistic action of the anterior cruciate ligament and thigh muscles in maintaining joint stability. Am J Sports Med. 1987;15(3):207‐213. doi:10.1177/036354658701500302 3618871

[7] Shultz SJ , Carcia CR , Perrin DH . Knee joint laxity affects muscle activation patterns in the healthy knee. J Electromyogr Kinesiol. 2004;14(4):475‐483. doi:10.1016/j.jelekin.2003.11.001 15165597

[8] Fleming BC , Renstrom PA , Ohlen G , et al. The gastrocnemius muscle is an antagonist of the anterior cruciate ligament. J Orthop Res. 2001;19(6):1178‐1184. doi:10.1016/S0736-0266(01)00057-2 11781021

[9] MacWilliams BA , Wilson DR , DesJardins JD , Romero J , Chao EY . Hamstrings cocontraction reduces internal rotation, anterior translation, and anterior cruciate ligament load in weight‐bearing flexion. J Orthop Res. 1999;17(6):817‐822. doi:10.1002/jor.1100170605 10632447

[10] Shelburne KB , Torry MR , Pandy MG . Muscle, ligament, and joint‐contact forces at the knee during walking. Med Sci Sports Exerc. 2005;37(11):1948‐1956. doi:10.1249/01.mss.0000180404.86078.ff 16286866

[11] Torry MR , Decker MJ , Ellis HB , Shelburne KB , Sterett WI , Steadman JR . Mechanisms of compensating for anterior cruciate ligament deficiency during gait. Med Sci Sports Exerc. 2004;36(8):1403‐1412. doi:10.1249/01.mss.0000135797.09291.71 15292750

[12] Williams GN , Snyder‐Mackler L , Barrance PJ , Buchanan TS . Quadriceps femoris muscle morphology and function after ACL injury: a differential response in copers versus non‐copers. J Biomech. 2005;38(4):685‐693. doi:10.1016/j.jbiomech.2004.04.004 15713288

[13] Kanno N , Amimoto H , Hara Y , et al. In vitro evaluation of the relationship between the semitendinosus muscle and cranial cruciate ligament in canine cadavers. Am J Vet Res. 2012;73(5):672‐680. doi:10.2460/ajvr.73.5.672 22533399

[14] Ramirez JM , Lefebvre M , Böhme B , Laurent C , Balligand M . Preactivation of the quadriceps muscle could limit cranial tibial translation in a cranial cruciate ligament deficient canine stifle. Res Vet Sci. 2015;98:115‐120. doi:10.1016/j.rvsc.2014.11.012 25487559

[15] Li G , Rudy TW , Sakane M , Kanamori A , Ma CB , Woo SL . The importance of quadriceps and hamstring muscle loading on knee kinematics and in‐situ forces in the ACL. J Biomech. 1999;32(4):395‐400. doi:10.1016/s0021-9290(98)00181-x 10213029

[16] Griffith CJ , Laprade RF , Coobs BR , Olson EJ . Anatomy and biomechanics of the posterolateral aspect of the canine knee. J Orthop Res. 2007;25(9):1231‐1242. doi:10.1002/jor.20422 17503521

[17] Victor J , Labey L , Wong P , Innocenti B , Bellemans J . The influence of muscle load on tibiofemoral knee kinematics. J Orthop Res. 2010;28(4):419‐428. doi:10.1002/jor.21019 19890990

[18] Kim SE , Jones SC , Lewis DD , et al. In‐vivo three‐dimensional knee kinematics during daily activities in dogs. J Orthop Res. 2015;33(11):1603‐1610. doi:10.1002/jor.22927 25982776

[19] Ichinohe T , Kanno N , Harada Y , Fujita Y , Fujie H , Hara Y . Analysis of passive tibio‐femoral joint movement of beagle dogs during flexion in cadaveric hind limbs without muscle. J Vet Med Sci. 2020;82(2):148‐152. doi:10.1292/jvms.18-0501 31839649 PMC7041994

[20] Riegert S . Anatomische und biomechanische Untersuchungen am Kniegelenk (Articulatio genus) des Hundes (Canis familiaris). Ludwig‐Maximilians‐Univ München. 2004. doi:10.5282/edoc.2637

[21] Arnoczky SP , Marshall JL . The cruciate ligaments of the canine stifle: an anatomical and functional analysis. Am J Vet Res. 1977;38(11):1807‐1814. 931164

[22] Tinga S , Kim SE , Banks SA , et al. Femorotibial kinematics in dogs with cranial cruciate ligament insufficiency: a three‐dimensional in‐vivo fluoroscopic analysis during walking. BMC Vet Res. 2018;14(1):85. doi:10.1186/s12917-018-1395-2 29530093 PMC5848543

[23] Knight RC , Thomson DG , Danielski A . Surgical management of pivot‐shift phenomenon in a dog. J Am Vet Med Assoc. 2017;250(6):676‐680. doi:10.2460/javma.250.6.676 28263119

[24] Husi B , Park B , Lampart M , Evans R , Pozzi A . Comparative kinetic and kinematic evaluation of TPLO and TPLO combined with extra‐articular lateral augmentation: a biomechanical study. Vet Surg. 2023;52(5):686‐696. doi:10.1111/vsu.13955 37011040

[25] Lampart M , Park BH , Husi B , Evans R , Pozzi A . Evaluation of the accuracy and intra‐ and interobserver reliability of three manual laxity tests for canine cranial cruciate ligament rupture‐an ex vivo kinetic and kinematic study. Vet Surg. 2023;52(5):704‐715. doi:10.1111/vsu.13957 37144831

[26] Widmer J , Cornaz F , Scheibler G , Spirig JM , Snedeker JG , Farshad M . Biomechanical contribution of spinal structures to stability of the lumbar spine‐novel biomechanical insights. Spine J. 2020;20(10):1705‐1716. doi:10.1016/j.spinee.2020.05.541 32474224

[27] Beer P , Knell SC , Pozzi A , Park BH . Biomechanical comparison of ex vivo lumbar vertebral fracture luxations stabilized with tension band or polymethylmethacrylate in cats. Vet Surg. 2020;49(8):1517‐1526. doi:10.1111/vsu.13516 32997834

[28] Warzee CC , Dejardin LM , Arnoczky SP , Perry RL . Effect of tibial plateau leveling on cranial and caudal tibial thrusts in canine cranial cruciate‐deficient stifles: an in vitro experimental study. Vet Surg. 2001;30(3):278‐286. doi:10.1053/jvet.2001.21400 11340560

[29] Noyes FR , Huser LE , West J , Jurgensmeier D , Walsh J , Levy MS . Two different knee rotational instabilities occur with anterior cruciate ligament and anterolateral ligament injuries: a robotic study on anterior cruciate ligament and extra‐articular reconstructions in restoring rotational stability. Arthroscopy. 2018;34(9):2683‐2695. doi:10.1016/j.arthro.2018.04.023 30173809

[30] Reif U , Hulse DA , Hauptman JG . Effect of tibial plateau leveling on stability of the canine cranial cruciate‐deficient stifle joint: an in vitro study. Vet Surg. 2002;31(2):147‐154. doi:10.1053/jvet.2002.31041 11884960

[31] Shahar R , Banks‐Sills L . Biomechanical analysis of the canine hind limb: calculation of forces during three‐legged stance. Vet J. 2002;163(3):240‐250. doi:10.1053/tvjl.2001.0660 12090766

[32] Apelt D , Pozzi A , Marcellin‐Little DJ , Kowaleski MP . Effect of cranial tibial closing wedge angle on tibial subluxation: an ex vivo study. Vet Surg. 2010;39(4):454‐459. doi:10.1111/j.1532-950X.2010.00670.x 20345522

[33] Tashman S , Anderst W , Kolowich P , Havstad S , Arnoczky S . Kinematics of the ACL‐deficient canine knee during gait: serial changes over two years. J Orthop Res. 2004;22(5):931‐941. doi:10.1016/j.orthres.2004.01.008 15304262

[34] Mengarelli A , Gentili A , Strazza A , Burattini L , Fioretti S , Di Nardo F . Co‐activation patterns of gastrocnemius and quadriceps femoris in controlling the knee joint during walking. J Electromyogr Kinesiol. 2018;42:117‐122. doi:10.1016/j.jelekin.2018.07.003 30025300

[35] Adrian CP , Haussler KK , Kawcak C , et al. The role of muscle activation in cruciate disease. Vet Surg. 2013;42(7):765‐773. doi:10.1111/j.1532-950X.2013.12045.x 23980704

[36] Andriacchi TP , Mündermann A , Smith RL , Alexander EJ , Dyrby CO , Koo S . A framework for the in vivo pathomechanics of osteoarthritis at the knee. Ann Biomed Eng. 2004;32(3):447‐457. doi:10.1023/b:abme.0000017541.82498.37 15095819

[37] Tashman S , Collon D , Anderson K , Kolowich P , Anderst W . Abnormal rotational knee motion during running after anterior cruciate ligament reconstruction. Am J Sports Med. 2004;32(4):975‐983. doi:10.1177/0363546503261709 15150046

[38] Blasimann A , Busch A , Henle P , Bruhn S , Vissers D , Baur H . Neuromuscular control in males and females 1 year after an anterior cruciate ligament rupture or reconstruction during stair descent and artificial tibial translation. Sci Rep. 2023;13(1):15316. doi:10.1038/s41598-023-42491-6 37714980 PMC10504317

[39] Zunzarren G , Garet B , Vinciguerra B , Murgier J . Persistence of neuromuscular activation deficit in the lower limb at 3‐years of follow‐up after ACL reconstruction surgery. Knee. 2023;43:97‐105. doi:10.1016/j.knee.2023.06.006 37385113

[40] Torres SJ , Nelson TJ , Pham N , Uffmann W , Limpisvasti O , Metzger MF . Suture tape augmentation increases the time‐zero stiffness and strength of anterior cruciate ligament grafts: a cadaveric study. Arthrosc Sports Med Rehabil. 2022;4(4):e1253‐e1259. doi:10.1016/j.asmr.2022.02.008 36033200 PMC9402422

[41] Wicks ED , Stack J , Rezaie N , Zeini IM , Osbahr DC . Biomechanical evaluation of suture tape internal brace reinforcement of soft tissue allografts for ACL reconstruction using a porcine model. Orthop J Sports Med. 2022;10(5):23259671221091252. doi:10.1177/23259671221091252 35547611 PMC9083057

[42] Wilson WT , Kennedy MJ , MacLeod D , Hopper GP , MacKay GM . Outcomes of anterior cruciate ligament reconstruction with independently tensioned suture tape augmentation at 5‐year follow‐up. Am J Sports Med. 2023;51(14):3658‐3664. doi:10.1177/03635465231207623 37975527 PMC10691290

