# Supplementary material for: The role of muscle forces on rotational and cranio‐caudal stability in the intact and CCL‐deficient stifle: An ex vivo biomechanical study
Source: Vet Surg. 2026 Jun 18;55(6):1067–77. doi: 10.1111/vsu.70122 (PMC13420933; doi:10.1111/vsu.70122)
Supplement: Supplementary file 1 — Table S1. Tibial internal rotation in degrees for the intact and CCL‐deficient stifle (±SD) at different muscle groups and activation levels with increments of 20% BW. Table S2. Mean CCT in the intact and CCL‐deficient stifle (±SD) at graded muscle activation increments of 20% BW. [file VSU-55-1067-s001.docx]

Supporting information

Table S1: Tibial internal rotation in degrees for the intact and CCL-deficient stifle (±SD) at different muscle groups and activation levels with increments of 20% BW. For each limb, muscle forces were applied at seven activation levels—0, 20, 30, 40, 60, 80, and 100 % of body weight (BW). “Q” = quadriceps, “G” = gastrocnemius, “B” = biceps femoris, “QG” = quadriceps + gastrocnemius, “QB” = quadriceps + biceps femoris, “BG” = biceps femoris + gastrocnemius, “QBG” = all three muscles. Values are presented separately for the intact and the CCL‑deficient stifle configurations; the intact values serve as the baseline control for each specimen. The final row (“Difference at 100 % BW”) lists the absolute difference in rotation between intact and CCL-deficient stifles at full muscle activation. (* indicates significant difference, Bonferroni adjustments were applied).

| Intact stifle | | | | | | | |
| --- | --- | --- | --- | --- | --- | --- | --- |
| Muscle activation (%BW) | Internal rotation (°) | | | | | | |
|  | Q | QB | B | BG | G | QG | QBG |
| 0 | 27.9  $\pm$10.8 | 27.9  $\pm$10.8 | 27.9  $\pm$10.8 | 27.9  $\pm$10.8 | 27.9  $\pm$10.8 | 27.9  $\pm$10.8 | 27.9  $\pm$10.8 |
| 20 | 24.6  $\pm$10.3 | 16.1  $\pm$6.7 | 24.2  $\pm$10.4 | 19.7  $\pm$9.0 | 24.4  $\pm$10.0 | 20.2  $\pm$8.5 | 12.4  $\pm$5.9 |
| 30 | 22.6  $\pm$10.4 | 12.3  $\pm$5.4 | 22.7  $\pm$10.5 | 16.4  $\pm$8.7 | 21.7  $\pm$8.9 | 16.6  $\pm$7.5 | 9.3  $\pm$4.9 |
| 40 | 19.7  $\pm8.4$ | 10.2  $\pm$4.5 | 19.3  $\pm$9.4 | 11.5  $\pm$6.4 | 19.6  $\pm$8.1 | 13.7  $\pm$5.9 | 8.1  $\pm$4.7 |
| 60 | 17.8  $\pm7.4$ | 9.1  $\pm$4.4 | 16.1  $\pm$8.8 | 9.9  $\pm$6.2 | 17.2  $\pm$7.7 | 10.6  $\pm$4.8 | 5.8  $\pm$2.8 |
| 80 | 16.1  $\pm$7.3 | 7.7  $\pm$3.8 | 14.0  $\pm$8.3 | 7.5  $\pm$4.5 | 14.7  $\pm$7.3 | 8.8  $\pm4$.4 | 5.5  $\pm$3.5 |
| 100 | 14.9  $\pm$6.6 | 6.8  $\pm$3.7 | 9.9  $\pm$6.6 | 5.8  $\pm$2.8 | 13.2  $\pm$7.1 | 7.6  $\pm$3.9 | 4.3  $\pm$2.3 |
| CCL-deficient stifle | | | | | | | |
| Muscle activation (%BW) | Internal rotation (°) | | | | | | |
|  | Q | QB | B | BG | G | QG | QBG |
| 0 | 34.8  $\pm$11.8 | 34.8  $\pm$11.8 | 34.8  $\pm$11.8 | 34.8  $\pm$11.8 | 34.8  $\pm$11.8 | 34.8  $\pm$11.8 | 34.8  $\pm$11.8 |
| 20 | 22.8  $\pm$9.5 | 13.7  $\pm$6.4 | 19.0  $\pm$8.4 | 17.4  $\pm$9.3 | 19.1  $\pm$9.9 | 15.5  $\pm$6.1 | 9.9  $\pm$4.1 |
| 30 | 20.20  $\pm$8.5 | 11.8  $\pm$6.8 | 16.1  $\pm$8.7 | 11.0  $\pm$7.1 | 18.0  $\pm$9.4 | 12.3  $\pm$5.9 | 6.6  $\pm$3.2 |
| 40 | 18.4  $\pm$7.8 | 8.7  $\pm$4.6 | 14.9  $\pm$8.1 | 8.6  $\pm$5.7 | 16.3  $\pm$8.3 | 10.4  $\pm$5.1 | 5.4  $\pm$2.6 |
| 60 | 15.7  $\pm$6.6 | 7.2  $\pm$3.4 | 12.6  $\pm$7.1 | 5.5  $\pm$2.4 | 13.5  $\pm$7.2 | 8.1  $\pm$4.2 | 4.2  $\pm$2.1 |
| 80 | 14.6  $\pm$6.9 | 5.7  $\pm$2.6 | 10.5  $\pm$6.3 | 4.4  $\pm$1.8 | 11.2  $\pm$5.6 | 6.0  $\pm$3.4 | 3.2  $\pm$1.5 |
| 100 | 11.8  $\pm$5.8 | 4.7  $\pm$2.4 | 8.1  $\pm$4.9 | 3.7  $\pm$1.5 | 9.7  $\pm$4.8 | 5.3  $\pm$3.0 | 2.8  $\pm$1.3 |
|  | | | | | | | |
| Difference at 100% BW  (Intact vs. CCL-deficient) | -3.1°,  -20.8% | -2.1°,  -30.9% | -1.8°,  -18.2% | -2.1°,  -36.2% | -3.5°,  -26.5% | -2.3°,  -30.3% | -1.5°,  -34.9% |

Table S2: Mean CCT in the intact and CCL-deficient stifle (±SD) at graded muscle activation increments of 20% BW. CCT was recorded at seven activation levels—0, 20, 30, 40, 60, 80, and 100 % body weight (BW)—following the loading sequence described in Table 1. Values are presented in paired columns (intact vs CCL-deficient stifles) for each activation step. The final three lines list the percent‑difference in CCT between CCL-deficient and intact stifles at baseline (0 % BW), at 20 % BW, and at 100 % BW, respectively. An asterisk denotes activation levels where CCT in the CCL-deficient stifle differs significantly from its intact counterpart (p < 0.05). (* indicates significant difference, Bonferroni adjustments were applied).

| Intact stifle | | | | | | | |
| --- | --- | --- | --- | --- | --- | --- | --- |
| Muscle activation  (% BW) | CCT in mm | | | | | | |
|  | Q | QB | B | BG | G | QG | QBG |
| 0 | 3.4  $\pm$0.5 | 3.4  $\pm$0.5 | 3.4  $\pm$0.5 | 3.4  $\pm$0.5 | 3.4  $\pm$0.5 | 3.4  $\pm$0.5 | 3.4  $\pm$0.5 |
| 20 | 3.14  $\pm$0.7 | 3.3  $\pm$0.6 | 3.3  $\pm$0.6 | 3.0  $\pm$0.6 | 3.1  $\pm$0.8 | 3.3  $\pm$0.9 | 3.2  $\pm$0.8 |
| 30 | 3.1  $\pm$0.6 | 3.2  $\pm$0.5 | 3.2  $\pm$0.6 | 3.0  $\pm$0.7 | 2.9  $\pm$0.6 | 3.1  $\pm$0.9 | 3.4  $\pm$0.8 |
| 40 | 3.0  $\pm$0.6 | 3.0  $\pm$0.5 | 3.1  $\pm$0.7 | 2.6  $\pm$0.8 | 2.9  $\pm$0.6 | 3.1  $\pm$0.7 | 3.3  $\pm$0.8 |
| 60 | 2.8  $\pm$0.6 | 2.6  $\pm$0.5 | 2.8  $\pm$0.6 | 2.5  $\pm$0.8 | 2.8  $\pm$0.6 | 3.2  $\pm$0.7 | 3.0  $\pm$0.6 |
| 80 | 2.6  $\pm$0.6 | 2.4  $\pm$0.4 | 2.7  $\pm$0.6 | 2.3  $\pm$0.6 | 2.6  $\pm$0.7 | 3.2  $\pm$0.6 | 3.2  $\pm$0.6 |
| 100 | 2.4  $\pm$0.6 | 2.2  $\pm$0.6 | 2.6  $\pm$0.7 | 2.3  $\pm$0.8 | 2.5  $\pm$0.6 | 3.0  $\pm$0.8 | 2.8  $\pm$0.5 |
| CCLD-deficient stifle | | | | | | | |
| Muscle activation (%BW) | CCT in mm | | | | | | |
|  | Q | QB | B | BG | G | QG | QBG |
| 0 | 25.2  $\pm$2.4 | 25.2  $\pm$2.4 | 25.2  $\pm$2.4 | 25.2  $\pm$2.4 | 25.2  $\pm$2.4 | 25.2  $\pm$2.4 | 25.2  $\pm$2.4 |
|  |  |  |  |  |  |  |  |
| 20 | 21.7  $\pm$2.7 | 19.2  $\pm$1.4 | 20.0  $\pm$2.0 | 19.5  $\pm$1.9 | 19.1  $\pm$2.4 | 18.6  $\pm$1.3 | 19.3  $\pm$2.3 |
|  |  |  |  |  |  |  |  |
| 30 | 20.1  $\pm$1.7 | 18.5  $\pm$1.6 | 19.4  $\pm$1.8 | 18.9  $\pm$1.8 | 18.4  $\pm$1.0 | 18.5  $\pm$1.4 | 19.2  $\pm$1.4 |
|  |  |  |  |  |  |  |  |
| 40 | 19.6  $\pm$1.4 | 18.3  $\pm$1.5 | 19.1  $\pm$1.3 | 18.7  $\pm$2.0 | 18.4  $\pm$1.4 | 18.5  $\pm$1.3 | 19.1  $\pm$1.2 |
| 60 | 19.3  $\pm$1.4 | 17.9  $\pm$1.4 | 18.3  $\pm$1.1 | 18.7  $\pm$1.9 | 18.2  $\pm$1.3 | 18.7  $\pm$1.3 | 19.2  $\pm$1.8 |
| 80 | 18.6  $\pm$1.7 | 17.8  $\pm$1.3 | 17.9  $\pm$1.4 | 18.8  $\pm$2.0 | 18.4  $\pm$1.3 | 19.0  $\pm$1.6 | 19.1  $\pm$1.3 |
| 100 | 18.4  $\pm$1.6 | 17.7  $\pm$1.4 | 17.3  $\pm$1.7 | 18.4  $\pm$1.4 | 18.6  $\pm$1.4 | 18.8  $\pm$1.3 | 19.1  $\pm$1.2 |
|  | | | | | | | |
| Difference at 100% BW muscle activation (intact vs. CCL-deficient) | 16  mm  153.8%  * | 15.5 mm  155.8%  * | 14.7 mm  147.7%  * | 16.1 mm  155.6%  * | 16.1 mm  152.6%  * | 15.8 mm 144.9%  * | 16.3  mm  148.9%  * |
